# Involvement of Acid-Sensing Ion Channel 1b in the Development of Acid-Induced Chronic Muscle Pain

**DOI:** 10.3389/fnins.2019.01247

**Published:** 2019-11-22

**Authors:** Chu-Ting Chang, Sitt Wai Fong, Cheng-Han Lee, Yu-Chia Chuang, Shing-Hong Lin, Chih-Cheng Chen

**Affiliations:** ^1^Institute of Biomedical Sciences, Academia Sinica, Taipei, Taiwan; ^2^Taiwan Mouse Clinic, National Comprehensive Mouse Phenotyping and Drug Testing Center, Academia Sinica, Taipei, Taiwan; ^3^Department of Neurobiology, Dana-Farber Cancer Institute, Harvard Medical School, Boston, MA, United States

**Keywords:** ASIC, ASIC1b, ASIC3, mambalgin-1, fibromyalgia, pain

## Abstract

Acid-sensing ion channels (ASICs) are important acid sensors involved in neural modulation in the central nervous system and pain-associated tissue acidosis in the peripheral system. Among ASIC subtypes, ASIC1b is the most selectively expressed in peripheral sensory neurons. However, the role of ASIC1b is still elusive in terms of its functions and expression profile. In this study, we probed the role of ASIC1b in acid-induced muscle pain in *Asic1b*-knockout (*Asic1b*^–/–^) and *Asic1b-Cre* transgenic (*Asic1b*^*Cre*^) mice. We tested the effect of ASIC1b knockout in a mouse model of fibromyalgia induced by dual intramuscular acid injections. In this model, a unilateral acid injection to the gastrocnemius muscle induced transient bilateral hyperalgesia in wild-type (*Asic1b*^+^*^/^*^+^) but not *Asic1b*^–/–^ mice; a second acid injection, spaced 1 or 5 days apart, to the same muscle induced chronic hyperalgesia lasting for 4 weeks in *Asic1b*^+^*^/^*^+^ mice, but the duration of hyperalgesia was significantly shortened in *Asic1b*^–/–^ mice. Mambalgin-1, an ASIC1b-containing channel inhibitor that was mixed with acid saline at the first injection, dose-dependently blocked the acid-induced transient and chronic hyperalgesia in *Asic1b*^+^*^/^*^+^ mice. In contrast, psalmotoxin 1 (PcTx1), an ASIC1a-selective antagonist, had no effect on acid-induced transient or chronic hyperalgesia. We used whole-cell patch clamp recording to study the properties of acid-induced currents in ASIC1b-expressing dorsal root ganglia (DRG) neurons from *Asic1b*^*Cre*^-TdTomato reporter mice. Medium- to large-sized ASIC1b-expressing DRG neurons mainly exhibited an amiloride-sensitive ASIC-like biphasic current (*I*_ASIC_) in response to acid stimulation, whereas small- to medium-sized ASIC1b-expressing DRG neurons predominantly exhibited an amiloride-insensitive sustained current. Specifically, mambalgin-1 selectively inhibited the *I*_ASIC_ in most ASIC1b-expressing DRG neurons. However, PcTx1 or APETx2 (an ASIC3-selective antagonist) had only a mild inhibitory effect on *I*_ASIC_ in about half of the ASIC1b-expressing DRG neurons. *In situ* hybridization revealed that ASIC1b-positive DRG neurons co-expressed highly with ASIC1a and ASIC2a mRNA and partially with ASIC3 and ASIC2b. Thus, ASIC1b might form a wide variety of heteromeric channels. ASIC1b-containing heteromeric channels might be promising targets for the therapeutic treatment of acid-induced chronic muscle pain.

## Introduction

Acid-sensing ion channels (ASICs) are proton-gated, voltage-independent and amiloride-sensitive sodium channels widely distributed in the central and peripheral nervous systems (Wemmie et al., 2013; Baron and Lingueglia, 2015). Because ASICs can be activated in physiological pH ranges, ASICs seem to be the fine acid sensors involved in acidosis-induced muscle pain (Immke and McCleskey, 2001; Birdsong et al., 2010; Reimers et al., 2017). Reduced pH value in muscle tissue elicited by ischemic myalgia, inflammation, lesions, tumors or fatiguing exercise results in ASIC activation and nociceptive transduction (Cheng et al., 2018; Lin et al., 2018).

There are at least 4 genes, encoding 6 polypeptides (subtypes), in the ASIC family — ASIC1a (Waldmann et al., 1997b), ASIC1b (Chen et al., 1998), ASIC2a (Waldmann et al., 1996), ASIC2b (Lingueglia et al., 1997), ASIC3 (Waldmann et al., 1997a), and ASIC4 (Akopian et al., 2000; Grunder et al., 2000). Each ASIC subtype varies widely in pH sensitivity, activation, inactivation kinetics, ion selectivity and pharmacology (Hesselager et al., 2004; Baron and Lingueglia, 2015; Cheng et al., 2018). A functional ASIC channel requires three polypeptides to form a homo- or heterotrimeric channel (Jasti et al., 2007; Cheng et al., 2018). Thus, revealing the molecular identity of ASIC composition and the roles of individual subtypes in somatosensory neurons would help in elucidating the contribution of ASICs in acidosis-induced muscle pain and reveal a more promising therapeutic target for the clinical treatment of muscle pain.

The involvement of ASIC3 in pain associated with tissue acidosis has been well characterized in many animal models (Wu et al., 2012; Lin et al., 2015; Hsu et al., 2019). In Sluka et al. (2001) developed a rodent model of chronic widespread muscle pain induced by dual intramuscular acid injections. The first acid injection to a unilateral muscle would induce bilateral, transient mechanical hyperalgesia that decreased in 24 h, whereas a second acid injection to the same muscle 5 days later would induce bilateral mechanical hyperalgesia lasting for 4 weeks. ASIC3 but not ASIC1a is involved in acid-induced mechanical hyperalgesia in muscle (Sluka et al., 2003). However, the involvement of other ASIC subtypes in the acid-induced muscle pain is not known.

ASIC1b is an acid sensor predominantly expressed in peripheral sensory neurons (Lin et al., 2015). Although ASIC1b was first identified almost two decades ago (Chen et al., 1998; Bassler et al., 2001), the exact neuronal subtype expressing ASIC1b and the role of ASIC1b in nociception are largely unknown. The role of ASIC1b-containing channels in inflammatory and neuropathic pain has been revealed by using pharmacological blockade by mambalgin-1, a 57-amino peptide isolated from mamba snake venom, which selectively inhibits both ASIC1a and ASIC1b activity with an IC_50_ of ∼11 to 250 nM (Diochot et al., 2012, 2016). Nevertheless, the identity of ASIC1b-containing channels involved in pain development is not known.

In this study, we aimed to examine the role of ASIC1b in sensory neurons related to acid-induced muscle pain. By generating *Asic1b*-knockout mice, we tested the effect of *Asic1b* knockout on acid-induced hyperalgesia with the dual intramuscular acid-injection model. Also, we generated *Asic1b-Cre* transgenic mice crossed with TdTomato protein reporter mice to examine the acid-induced currents and the co-localization of ASIC1b with other ASIC subtypes in ASIC1b-expressing dorsal root ganglia (DRG) neurons.

## Materials and Methods

### Animals

Mice with 8 to 12 weeks old were used in all experiments. *Asic1b* wild-type (*Asic1b*^+^*^/^*^+^) and knockout (*Aisc1b*^–/–^) mice were offspring of *Asic1b*^±^ mice bred in a congenic C57BL6/J background. All procedures were approved by the Institutional Animal Care and Utilization Committee of Academia Sinica.

### Generation of ASIC1b-Cre Transgenic Mice

**FIGURE 1:**
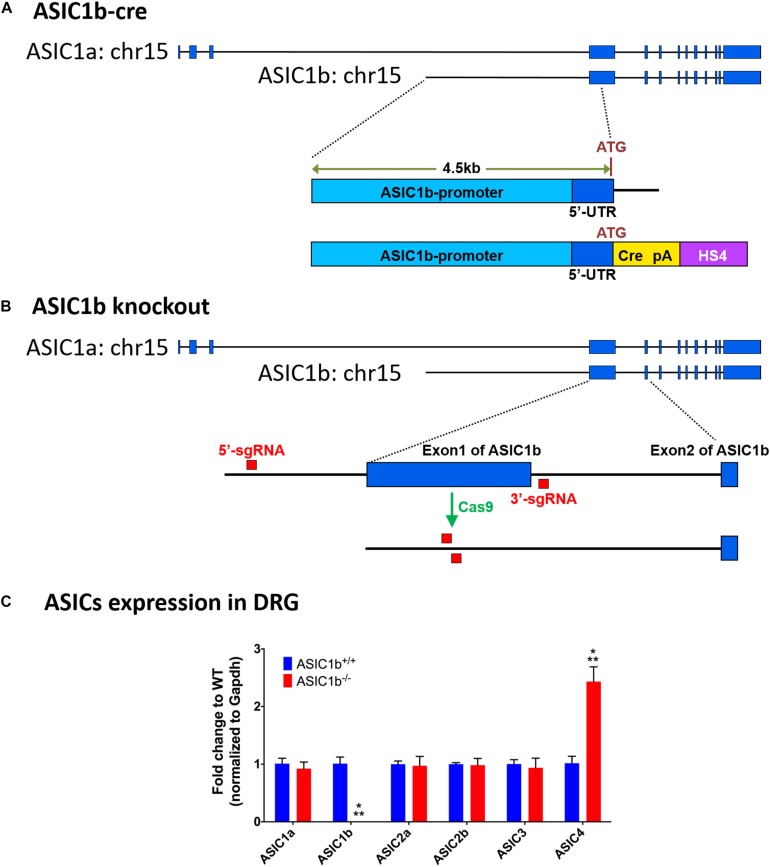
Experimental design of generation of *Asic1b-Cre* transgenic and *Asic1b* knockout mice. **(A)** Generation of *Asic1b-Cre* mice was based on a 4.5 Kb Asic1b promoter DNA with an insertion of the Cre-polyA-HS4 cassette right after ATG codon. **(B)** Experimental design of *Asic1b* knockout via CRISPR/Cas9 technology. Two single-guide RNAs (sgRNAs) were used to target the 5′-upstream of exon 1 and the 3′-downstream of exon 1 of Asic1b. **(C)** The gene expression of ASIC subtypes in the lumbar part of DRG of *Asic1b*^+^*^/^*^+^ and *Asic1b*^–/–^ mice (*N* = 3). The gene expression levels were analyzed by quantitative real-time polymerase chain reaction. Compared with the *Asic1b*^+^*^/^*^+^, lower expression of Asic1b was detected in the DRG of *Asic1b*^–/–^ mice. For the expression of Asic1a, Asic2a, Asic2b, Asic3 transcripts, no difference was detected between *Asic1b*^+^*^/^*^+^ and *Asic1b*^– /–^ mice in the DRG. The expression of Asic4 was very low in DRG, although it showed >2-fold difference between *Asic1b*^+^*^/^*^+^ and *Asic1b*^– /–^. With normalized with gapdh mRNA, the ΔCT mean of ASIC4 of *Asic1b*^+^*^/^*^+^ mouse was 10.14 ± 0.32 and of *Asic1b*^– /–^ was 8.88 ± 0.25, while one of Asic3 of *Asic1b*^+^*^/^*^+^ mouse was 4.76 ± 0.19. ^∗∗∗^*P* < 0.001 vs. *Asic1b*^+^*^/^*^+^.

To target the DRG neurons expressing ASIC1b, we used a ∼4.5-kb genomic (129/SvJ) DNA fragment, upstream of the ATG translation start codon of ASIC1b, to drive Cre recombinase with a poly A signal (Figure 1A). The whole ASIC1b-Cre DNA cassette was flanked by the chicken hypersensitive site 4 (HS4) insulator provided by Dr. Ching-Yen Tsai (Transgenic Core Facility of the Institute of Molecular Biology, Academia Sinica Taiwan). High-quality linearized plasmid DNA was used to generate the *Asic1b-Cre* (*Asic1b*^*Cre*^) mouse lines by pronuclear microinjection in C57/BL6 zygotes. Five founder mice bearing the Cre transgene were retained for copy number verification and two founder lines bearing a minimum copy transgene were screened for correct expression of Cre by crossing with the ROSA-Gt-LacZ Cre reporter. One of the five founder mice showing expression of Cre only in the trigeminal ganglia and DRG but not the brain was used for *in situ* hybridization validation. We used the following primers for genotyping:

Asic1b-cre WT-forward: 5′-CCAGCGGTGGGTCAGGAAT GGC-3′
Asic1b-cre WT-reverse: 5′-CCTCCGCTGGCCCGGCTGT TTG-3′
Asic1b-cre Cre-reverse: 5′-TGCGAACCTCATCACTCGT TGC-3′

*PCR program*: genomic DNA was amplified by first denaturing at 95°C for 10 min followed by 41 cycles of denaturing at 95°C for 30 s, annealing at 63°C for 35 s, and extension at 72°C for 90 s followed by a final extension at 72°C for 10 min.

### Generation of *Asic1b*-Knockout Mouse Line by CRISPR/Cas9 Technology

ASIC1a and ASIC1b are encoded by the *ACCN2* gene. They are different only in the 5′ terminus of the mRNA (exon 1-2 of *Asic1a* and exon 1 of *Asic1b*). We generated the *Asic1b*^–/–^ mouse line by direct application on mouse zygotes by using the genome-editing Clustered Regularly Interspaced Short Palindromic Repeats (CRISPR) technology. Briefly, we injected Cas9 RNA with two single-guide RNAs (sgRNAs), one targeting the 5′-upstream of exon 1 and another targeting the 3′-downstream of exon 1 of *Asic1b*, into the nucleus of mouse zygotes (Figure 1B). After oviduct embryo transfer, we screened for the correct *Asic1b* exon 1 deletion line by PCR with the following primers:

ASIC1b-WT forward: 5′-AGTTTCTGCTTGTGAATCCA GGT-3′
ASIC1b-WT reverse: 5′-TCAAAACATTGCAGATGTGA GGC-3′
ASIC1b-KO reverse: 5′-ACTCCAGCATAACACCTCTT CTC-3′

*PCR program*: genomic DNA was amplified by first denaturing at 95°C for 10 min followed by 41 cycles of denaturing at 95°C for 30 s, annealing at 60°C for 35 s, and extension at 72°C for 90 s followed by a final extension at 72°C for 10 min.

### RNA Extraction and Quantitative Real-Time Polymerase Chain Reaction (qRT-PCR)

The DRG samples of the lumbar parts were collected from 16-week-old female mice with *Asic1b*^+^*^/^*^+^ or *Asic1b*^–/–^ genotypes. RNA extraction of the DRG samples was conducted with RNeasy Mini Kit (Qiagen) with on column DNase digestion (Qiagen), based on the manufacturer’s protocol. The RNA concentration and quality were measured by NanoDrop 1000 (Thermo Scientific). The cDNA reverse transcribed from RNA was prepared using with the iScript cDNA synthesis kit (Bio-Rad). Gene expression of DRG was measured using FastStart Universal SYBR Green Master with Rox (Roche) and analyzed using ABI 7500 Real Time PCR System (Life Technologies). Gene expression was normalized to gapdh mRNA. Data are presented as fold-change of gene expression in each group relative to the WT group of each gene. The primers of ASIC1a, ASIC1b, ASIC2a, ASIC2b, ASIC3, ASIC4, and gapdh were adapted from the paper of Wu et al. (2019).

**Table:** 

**Gene**	**Access No.**	**primer**	
ASIC1a	NM_009597	forward	GAACTGAAGACCGAGGAGGAG
		reverse	GCCGCTCATAGGAGAAGATGT
ASIC1b	NM_001289791	forward	TCAGCTACCCTGACTTGCTCTA
		reverse	GAGCGGTTGTAGAAACGATGGA
ASIC2a	NM_001034013	forward	CGATGGACCTCAAGGAGAGC
		reverse	ATACACGAAGATGTGGCGGAT
ASIC2b	NM_007384	forward	CTTGCTGTTGTCCTGGTCCT
		reverse	TTGTTGTTGCACACGGTGAC
ASIC3	NM_183000	forward	TATGTGGCTCGGAAGTGCGGAT
		reverse	CAGACACAAGTGTCCTTTCGCAG
ASIC4	NM_183022	forward	CACCTTGCTGGAGATCCTTGA
		reverse	GTCCGCAGTGGGGTCTTG
Gapdh	NM_001289726	forward	ATGTGTCCGTCGTGGATCTG
		reverse	CCTCAGTGTAGCCCAAGATG
			

#### *In situ* Hybridization

To collect DRG samples, mice were anesthetized with 1.3 mg/kg urethane (Sigma-Aldrich, St. Louis, MO, United States) by intraperitoneal injection, then perfused with 4% paraformaldehyde (Merck, Germany) in phosphate buffered saline (pH 7.4). For *in situ* hybridization, we used 12-μm-thick DRG cryosections mounted on VWR Microslides (VWR, United States) and the RNAscope^®^ fluorescent multiplex reagent kit (Advanced Cell Diagnostics, United States) according to the manufacturer’s instructions. The RNA probe for *in situ* hybridization was designed and provided by Advance Cell Diagnostics. For triple hybridization, the probes for ASIC1a (Cat. 462381-C1) targeting on region 178–749, ASIC1b (Cat. 474591-C2) targeting on region 476–1232 and ASIC3 (Cat. 480541-C3) targeting on region 284–1263 were combined. For duplex hybridization, we used ASIC1b (Cat. 474591-C1) targeting on region 476–1232 combined with ASIC2a (Cat. 480571-C3) targeting on region 2–762 or ASIC2b (Cat. 489031-C2) targeting on region 61–1132.

#### Pain Behavioral Assay

Mice under anesthesia with 2% isoflurane received a 20-μL injection of acid saline (pH 4.0, buffered with 1 mM 2-[N-morpholino]ethanesulfonic acid [MES]) into the left gastrocnemius muscle. Before the von Frey test, mice were habituated in a cage with stainless steel mesh covered with acrylic glass for 1 h. To assess mechanical hyperalgesia, an 0.2 mN von Frey filament was used to stimulate the plantar surface of both hind paws. We stimulated mice five times on each hind paw. The mechanical hyperalgesia was evaluated by the total withdrawal responses of both hind paws. A positive response was defined as lifting or flinching of the stimulated paw when a von Frey filament was applied.

#### Gastrocnemius Muscle Retrograde-Trace

Mice were anesthetized with 2% isoflurane and injected with 10 μL of 4% (wt/vol) fluorogold (Fluorochrome) into the gastrocnemius muscle of both legs. DRG were harvested at 5 to 7 days after injection.

#### DRG Primary Culture

Mice were killed with CO_2_, and lumbar DRGs were collected in Ca^2+^/Mg^2+^ free Hank’s balanced salt solution (HBSS, Thermo Fisher Scientific). The DRG samples were digested with 0.125% (wt/vol) collagenase IA (Sigma) for 70 min and 0.125% (vol/vol) trypsin (Thermo Fisher Scientific) for 15 min at 37°C in 1X HBSS. For each digestion, collagenase type IA was diluted with HBSS, then removed after centrifugation, and trypsin was neutralized with DMEM (Gibco) containing 10% (vol/vol) fetal bovine serum (FBS). Fully digested DRG neurons were triturated and plated on poly-L-lysine–coated cover slides. The DRG neurons were cultured in 3.5 cm petri dish with DMEM plus 10% (vol/vol) FBS (Gibco), 1% (vol/vol) penicillin/streptomycin (Gibco) and were maintained in a 5% CO2–95% O2 (vol/vol) incubator at 37°C.

#### Whole-Cell Patch-Clamp Recording

All recordings occurred within 30 h after seeding. Whole-cell patch-clamp recordings were performed with an Axopatch MultiClamp 700B (Axon Instruments) as previously described (Lin et al., 2012). All experiments were performed at room temperature (22–25°C). The pipette resistance was 6 to 10 MΩ.

#### Drugs and Solutions

The internal solution contained (in mM) 100 KCl, 2 Na2-ATP, 0.3 Na3-GTP, 10 EGTA, 5 MgCl2, and 40 Hepes adjusted to pH 7.4 with 1 M KOH. The osmolarity of internal solution was 290 to 300 mOsm/L. Coverslips were positioned in a chamber continuously perfused with artificial cerebrospinal fluid (ACSF) with gravitation force. The ACSF contained (in mM) 130 NaCl, 5 KCl, 1 MgCl2, 2 CaCl2, 10 glucose, and 20 Hepes adjusted to pH 7.4 with 5N NaOH. The osmolarity of ACSF was almost 300 mOsm/L.

Mambalgin-1, APETx2, and psalmotoxin 1 (PcTx1) were purchased from Alomone Labs and prepared by autoclaved water in stock solutions of 200 μM, 1 mM, and 200 μM respectively. Mambalgin-1, APETx2, and PcTx1 were diluted with ACSF to designated concentrations for electrophysiology experiments. Amiloride was purchased from Sigma and prepared at 100 mM by autoclaved water in stock solution and diluted with ACSF to 100 μM for experiments. Acidic ACSF was titrated with 1 M MES.

#### Action Potential (AP) Parameters

Action potential parameters were determined after entering the whole-cell condition, in which the neuron was held at −70 mV with bridge balance and received a 0.5-s 30-pA current step during a 3-s frame. The sampling rate was 50 kHz. All AP parameters were determined from the first spike when AP was evoked. The criterion of AP threshold was set at the slope about 20 because it was close to the point of a sharp upward increase. Rheobase was defined by the minimal current evoked the first spike during the 30-pA increase of the step. Then, a 2× Rheobase current was injected to determine the AP profile (single or multiple spikes).

#### ASIC Parameters

After AP parameters were determined, we switched to the voltage clamp mode to detect the acid-induced inward current. Series resistance was compensated by 70%. Sampling rate was 6.6 kHz and low-pass filter at 1 kHz. We puffed the acidic ACSF through a theta glass pipette 100 μm away from the recorded neuron and controlled by a VC-6 six-channel valve controller (Warner Instruments) via gravity and fast changed the solution by using a fast-stepper (Warner, SF-77C). After the acidic ACSF solution was applied to the recorded cell five times, we changed the acidic ACSF and pH 7.4 ACSF with that containing amiloride, APETx2, PcTx1, or mambalgin-1. A drug-sensitive current was defined, if the acid-induced current (in peak amplitude) was reduced >10% during drug application.

#### Statistical Analysis

Data are presented as mean ± SEM and were analyzed by using Prism 6. Mechanical withdrawal response and drug effects were tested by repeated measure two-way ANOVA followed by *post hoc* Sidak test. Area under the receiver operating characteristic curve (AUC; withdrawal response, days) after the second injection was calculated by the trapezoidal method. One-way ANOVA followed by *post hoc* Dunnett test was used to analyze the drug effect determined by the AUC after the second acid injection. *P* < 0.05 was considered statistically significant. Desensitization of the acid-induced inward current was fitted to the single exponential function with the following equation:

$$f\,\left(x\right)=a_{0}\,e^{-\frac{x}{\tau }}+a_{1}$$

## Results

**FIGURE 2:**
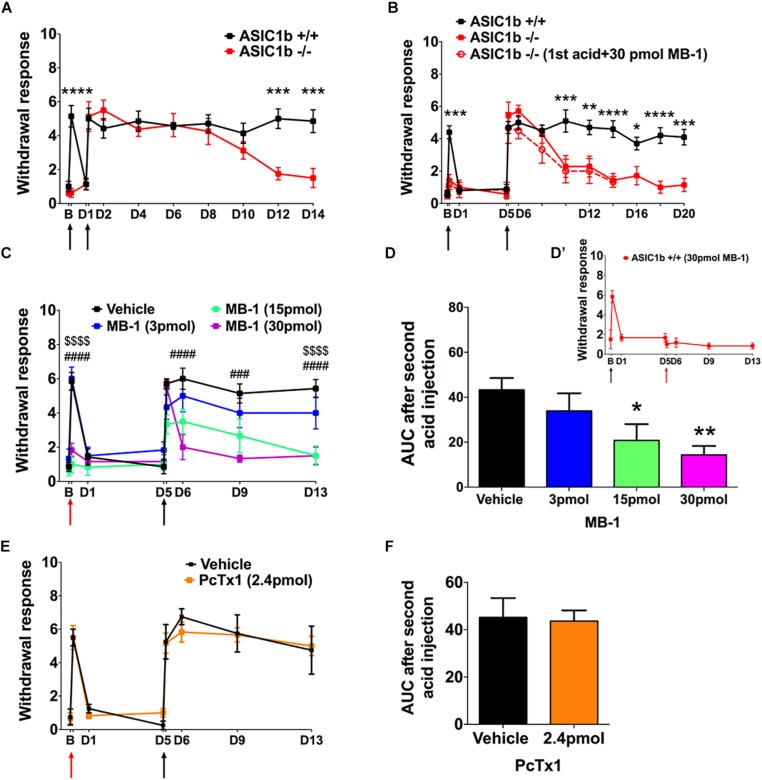
A role for ASIC1b in acid-induced mechanical hyperalgesia. **(A)** Mechanical hyperalgesia of wild-type (*Asic1b*^+^*^/^*^+^, *N* = 7) and ASIC1b-knockout (*Asic1b*^– /–^, *N* = 8) mice was induced by intramuscular injections of pH-4.0 saline on days 0 and 1 and evaluated by 0.20-mN von Frey filament. Data were analyzed by two-way ANOVA (Interaction *F*_(__10_,_143__)_ = 5.34, *P* < 0.0001; Time *F*_(__10_,_143__)_ = 14.5, *P* < 0.0001; Genotype *F*_(__1_,_143__)_ = 23.11, *P* < 0.0001), followed by Sidak *post hoc* test. ^∗∗∗^*P* < 0.001 for genotype difference at specific times. **(B)** Mechanical hyperalgesia of *Asic1b*^+^*^/^*^+^ (*N* = 10) and *Asic1b*^– /–^ (*N* = 7) mice was induced by intramuscular injections of pH-4.0 saline on days 0 and 5. Data were analyzed by two-way ANOVA [Interaction *F*_(__12_,_195__)_ = 5.933, *P* < 0.0001; Time *F*_(__12_,_195__)_ = 21.28, *P* < 0.0001; Genotype *F*_(__1_,_195__)_ = 59.07, *P* < 0.0001], followed by Sidak *post hoc* test. ^****^P < 0.0001; ^∗∗∗^*P* < 0.001; ^∗∗^*P* < 0.01; ^∗^*P* < 0.05 *Asic1b*^+^*^/^*^+^ vs. *Asic1b*^– /–^. For comparison, the effect of mambalgin-1 on acid-induced hyperalgesia in *Asic1b*^– /–^ mice was plotted. **(C)** Effect of mambalgin-1 (MB-1) on acid-induced mechanical hyperalgesia (vehicle, *N* = 7; MB-1 [3 pmol], *N* = 6; MB-1 [15 pmol], *N* = 7; MB-1 [30 pmol], *N* = 7). Data were analyzed by two-way ANOVA [Interaction *F*_(__21_,_168__)_ = 4.014, *P* < 0.0001; Time *F*_(__7_,_168__)_ = 26.61, *P* < 0.0001; Drug dose *F*_(__3_,_168__)_ = 29.32, *P* < 0.0001], followed by Sidak *post hoc* test. ^###^*P* < 0.001, ^####^*P* < 0.0001, MB-1 (30 pmol) vs. vehicle; ^$$$$^*P* < 0.0001, MB-1 (15 pmol) vs. vehicle. **(D)** Cumulative withdrawal response after second acid injection 5 days before acid + MB-1 injection is shown as the area under the receiver operating characteristic curve (AUC) calculated by the trapezoidal method. Data were analyzed by one-way ANOVA [*F*_(__3_,_21__)_ = 4.719, *P* = 0.0114], followed by Dunnett *post hoc* test. ^∗∗^*P* < 0.01, ^∗^*P* < 0.05 vs. vehicle. **(D’)** While 30 pmole mambalgin-1 was applied in the second acid injection, it blocked the development of the acid-induced chronic hyperalgesia. **(E)** Effect of PcTx1 on acid-induced mechanical hyperalgesia. Data were analyzed by two-way ANOVA (vehicle, *N* = 4; PcTx1 [120 nmol], *N* = 6) Interaction *F*_(__7_,_64__)_ = 0.2935, *P* = 0.9541; Time *F*_(__7_,_64__)_ = 31.29, *P* < 0.0001; Drug dose *F*_(__1_,_64__)_ = 0.054, *P* = 0.8169). **(F)** Cumulative withdrawal response after a second acid injection 5 days before acid + PcTx1 injection is shown as the AUC calculated by the trapezoidal method. Data were analyzed by unpaired *t*-test [*F*_(__3_,_5__)_ = 2.191, *P* = 0.4149). Black arrows, the time mice received intramuscular injection of pH 4.0 saline. Red arrows, the time mice received intramuscular injection of pH-4.0 saline mixed with mambalgin-1 or PcTx1. B, baseline. Data are mean ± SEM.

To evaluate the possible gene compensation effect in *Asic1b*^–/–^ mice, we conducted qRT-PCR to examine the gene expression level of ASICs in the lumbar part DRG neurons. We found no significant change of ASIC1a, ASIC2a, ASIC2b, and ASIC3 transcripts between *Asic1b*^+^*^/^*^+^ and *Asic1b*^–/–^ mice (Figure 1C). Also, the expression of ASIC1b transcript was not detectable in *Asic1b*^–/–^. To determine the role of ASIC1b in the acidosis-induced muscle pain, we used the dual acid injection model, a well-established model used to test acid-induced transient and chronic hyperalgesia, as well as hyperalgesic priming (Sluka et al., 2001; Chen and Chen, 2014; Chen et al., 2014). In wild-type mice, injecting dual pH-4.0 acidic saline 1 day apart into the unilateral gastrocnemius muscle induced bilateral transient mechanical hyperalgesia after the first acid injection, which declined at 24 h; bilateral chronic hyperalgesia developed after the second acid injection to the same muscle (Figure 2). In contrast, in *Asic1b*^–/–^ mice, transient hyperalgesia after the first acid injection was almost abolished; the duration of long-lasting hyperalgesia induced by the second acid injection was significantly shorter than that in wild-type littermates (*Asic1b*^+^*^/^*^+^) (Figure 2A). The duration of chronic hyperalgesia was even shorter in *Asic1b*^–/–^ mice when the dual acid injections were spaced 5 days apart (Figure 2B). Of note, the ASIC1-seletive antagonist mambalgin-1 showed no effect on acid-induced hyperalgesia in *Asic1b*^–/–^ mice. These results suggest that ASIC1b contributes to the acid-induced acute response (transient hyperalgesia) and also participates in the maintenance of long-lasting hyperalgesia.

We next used a pharmacological approach to investigate how ASIC1b-containing channels could be involved in the acid-induced hyperalgesia. Previous studies have demonstrated that mambalgin-1 selectively targets ASIC1b-containing channels to alleviate hyperalgesia in inflammatory and neuropathic pain models (Diochot et al., 2012, 2016), so we further examined the effect of mambalgin-1 in the dual acid injection model. We mixed different doses of mambalgin-1 with pH-4.0 acidic saline for the first acid injection and used only pH-4.0 acidic saline in the second injection 5 days later. The first acid-induced transient hyperalgesia was almost abolished at mambalgin-1 doses of 15–30 pmol, but the hyperalgesia responses induced by the second acid injection were greatly affected by mambalgin-1 dose-dependently (Figure 2C). We further examined the cumulative withdrawal response after the second injection by calculating the AUC. As compared with the vehicle control, with high doses of mambalgin-1 (15 and 30 pmol), the AUC significantly decreased after the second acid injection (Figure 2D). While mambalgin-1 (30 pmol) was applied in the second acid injection, it also inhibited the development of chronic hyperalgesia (Figure 2D’). However, the first acid injection mixed with PcTx1, a selective ASIC1a inhibitor at low doses, did not affect transient or chronic hyperalgesia in the same experimental protocol as mambalgin-1 (Figures 2E,F). The mambalgin-1 results agree with the effect of *Asic1b* knockout in the dual acid injection model and the effect of mambalgin-1 on selectively blocking ASIC1b-containing channels.

**FIGURE 3:**
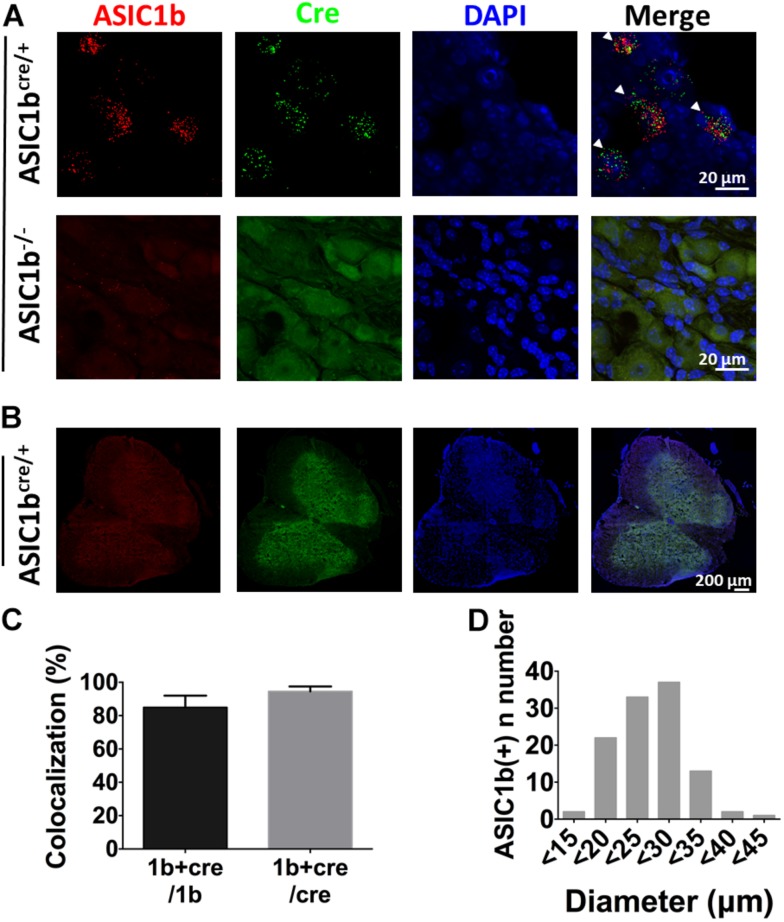
Validation of *Asic1b-Cre* and *Asic1b*-knockout mice by *in situ* hybridization. **(A)** Duplex hybridization of ASIC1b and Cre transcripts in L4 DRG neurons of *Asic1b*^*Cre/*+^ (arrowheads) and *Asic1b*^– /–^ mice. **(B)** Lack of ASIC1b and Cre transcripts in the spinal cord of *Asic1b*^*Cre/*+^ mice. **(C)** Quantitative analysis of *Asic1b* and *Cre* co-localization in *Asic1b*^*Cre/*+^ DRG neurons (*N* = 3 mice). Data are mean ± SEM. **(D)** Histogram of ASIC1b-expressing DRG neurons by size.

To further elucidate the expression profile and functionality of ASIC1b-containing channels in peripheral sensory neurons, we generated transgenic *Asic1b*^*Cre*^ mice to identify the ASIC1b-expressing neurons in DRG. To validate the *Asic1b*^*Cre*^ line, we used *in situ* hybridization to analyze the expression of ASIC1b and Cre transcripts in DRG and the spinal cord (Figure 3). *Asic1b* transcripts highly co-localized with Cre transcripts in *Asic1b*^*Cre/*+^ L4 DRG neurons, but both transcripts were absent in *Asic1b*^–/–^ DRG neurons (Figure 3A). Also, neither *Asic1b* nor *Cre* transcripts were detectable in the spinal cord of *Asic1b*^*Cre/*+^ mice (Figure 3B). Quantitative analyses showed that 84.9% of *Asic1b*-expressing neurons co-expressed *Cre* transcripts and 94.5% of *Cre*-expressing neurons co-expressed *Asic1b* transcripts (Figure 3C). The *Asic1b*-expressing neurons were distributed in a wide range of cell sizes (Figure 3D).

**FIGURE 4:**
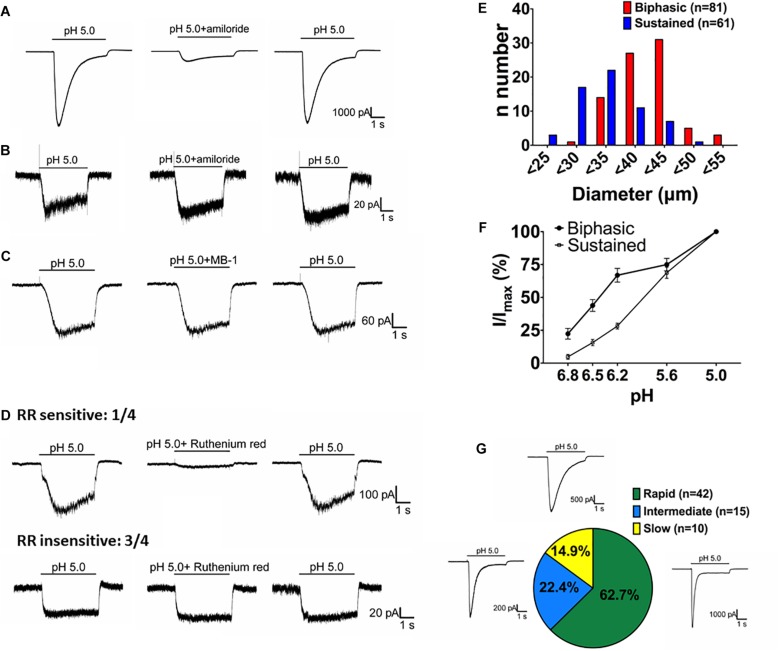
Acid-induced currents in ASIC1b-expressing DRG neurons. **(A)** Representative current traces of ASIC-like currents in ASIC1b-expressing DRG neurons. Amiloride (100 μM) inhibited the acid-induced transient inward current (*n* = 5). **(B)** Representative current traces of acid-induced sustained currents in ASIC1b-expressing DRG neurons (*n* = 5). **(C)** Representative current traces of acid-induced sustained currents resistant to mambalgin-1 (1 μM). **(D)** Representative current traces of acid-induced sustained currents sensitive (*n* = 1) or resistant (*n* = 3) to 10 μM ruthenium red treatment. **(E)** Histograms of DRG neurons by size and acid-induced current types (biphasic, *n* = 81; sustained, *n* = 61). **(F)** pH sensitivity of DRG neurons with biphasic and sustained currents. Peak amplitudes were normalized to that induced by pH-5 artificial cerebrospinal fluid (ACSF) (biphasic, *n* = 10; sustained, *n* = 10). **(G)** The proportion of three subtypes of ASIC-like currents with different desensitization time constants (rapid, <300 ms; intermediate, 300–900 ms; and slow, >900 ms).

By crossing *Asic1b^*Cre*/^*^+^ mice with TdTomato reporter mice, we could selectively target ASIC1b-expressing neurons based on the expression of TdTomato. We used whole-cell patch clamp recordings to analyze the acid-induced currents in ASIC1b-expressing DRG neurons and found that all were depolarized on stimulation with acid (pH 5.0) (Figure 4). ASIC1b-expressing DRG neurons can be characteristically divided into 2 different subtypes. In 142 ASIC1b-expressing DRG neurons, 57% (*n* = 81) expressed an amiloride-sensitive ASIC-like current, whereas the other 43% (*n* = 61) expressed an amiloride-resistance sustained inward current (Figures 4A,B). The amiloride-resistant current was also resistant to mambalgin-1 (Figure 4C) and its current amplitude was much lower than that of ASIC-like current (137.9pA vs. 3793pA) (Table 1). In a small portion of neurons, the acid-induced sustained inward current was inhibited by 10 μM ruthenium red, a non-selective blocker for TRP channels (Figure 4D; Jordt et al., 2000; Hamilton et al., 2016). ASIC1b was expressed in a wide range of cell-sized DRG neurons, from 20 to 55 μm in diameter (Figure 4E). Most medium- to large-sized (>30 μm) ASIC1b-expressing DRG neurons exhibited an ASIC-like current (*I*_ASIC_); whereas most small-sized (<30 μm) ASIC1b-expressing DRG neurons exhibited an acid-induced sustained current (Figure 4E). These 2 ASIC1b-expressing neuron subtypes showed different acid sensitivity, AP configurations, and many biophysical properties (Figure 4F and Table 1).

**TABLE 1:** Electrophysiological properties of two types of pH-5–evoked currents on ASIC1b-TdTomato(+) dorsal root ganglia (DRG) neurons.

	**Biphasic**	**Sustained**	**Biphasic vs. sustained**	**Test**
pH_50_	6.35 ± 0.07(10)	5.88 ± 0.04(10)	*P* < 0.0001	UPT
Current amplitude (pA)	3793 ± 293.4(68)	137.9 ± 15.3(45)	*P* < 0.0001	MW
AP threshold (mV)	−33.3 ± 0.8(81)	−28.1 ± 0.9(61)	*P* < 0.0001	MW
AP rheobase (pA)	631.2 ± 55.4(81)	337 ± 50.2(61)	*P* < 0.0001	MW
AP half-width (ms)	1.2 ± 0.1(81)	2.4 ± 0.2(61)	*P* < 0.0001	MW
AP overshoot (mV)	29.3 ± 2(81)	41.2 ± 1.9(61)	*P* < 0.0001	MW
Multiple spike (%)	6.7% (3/45)	33.3% (7/21)	*P* < 0.01	F
RMP (mV)	−56.8 ± 0.6(71)	−50.9 ± 1.1(59)	*P* < 0.001	MW
Cell size (μm)	39.4 ± 0.5(81)	32.7 ± 0.7(61)	*P* < 0.0001	UPT
Membrane capacitance (pF)	48.3 ± 3.4(84)	26.4 ± 2.7(67)	*P* < 0.0001	MW
Membrane resistance (mΩ)	241.1 ± 25.1(84)	475.8 ± 35.6(67)	*P* < 0.0001	MW
Membrane time constant (ms)	0.78 ± 0.05(84)	0.6 ± 0.03(67)	*P* < 0.01	MW

*Electrophysiological properties of ASIC1b-TdTomato(+) DRG neurons with biphasic and sustained pH-5–induced acid current. Data are mean ± SEM. UPT, unpaired *t*-test; MW, Mann-Whitney test; F, Fisher’s exact test; RMP, resting membrane potential.*

We divided the ASIC1b-expressing neurons with *I*_ASIC_ into three subgroups based on desensitization rate: rapid (τ_desens_ < 300 ms), intermediate (τ_desens_ 300−900 ms) and slow (τ_desens_ < 900 ms). In response to pH-5 acidic ACSF, 42 of 67 neurons (62.7%) showed rapid desensitization; 15 of 67 (22.4%) intermediate desensitization, and 10 of 67 (14.9%) slow desensitization (Figure 4G). Neurons with rapid desensitization showed lower threshold and higher RMP and Rheobase than neurons with intermediate and slow desensitization, although all three subgroups had similar AP configuration (Table 2).

**TABLE 2:** Electrophysiological properties of three types of ASIC1b-TdTomato(+) DRGs with different desensitization rates.

	**Rapid (*n* = 42) τ_desens_ < 300**	**Intermediate (*n* = 15) τ_desens_300−900**	**Slow (*n* = 10) τ_desens_ > 900**	**Rapid vs. intermediate**	**Rapid vs. slow**	**Intermediate vs. slow**	**Test**
AP threshold (mV)	−32.2 ± 1.2	−35.7 ± 4.7	−39.6 ± 1	NS	*P* < 0.01	NS	KW
AP rheobase (pA)	678 ± 501.2	404.6 ± 64.2	316.5 ± 105.6	NS	*P* < 0.01	NS	KW
AP half-width (ms)	1.3 ± 0.9	0.9 ± 0.1	0.8 ± 0.1	NS	NS	NS	KW
AP overshoot (mV)	33.7 ± 2.6	17.5 ± 3.5	24.5 ± 4	*P* < 0.01	NS	NS	ANOVA
RMP (mV)	−58.9 ± 0.6	−51.9 ± 1	−51.9 ± 0.8	*P* < 0.0001	*P* < 0.0001	NS	ANOVA
Cell size (μm)	39 ± 0.7	38 ± 1.5	40.9 ± 1.4	NS	NS	NS	ANOVA
Membrane capacitance (pF)	46.1 ± 4.9	34.3 ± 3.7	60.4 ± 9	NS	NS	NS	KW
Membrane resistance (mΩ)	248.1 ± 31.7	263.6 ± 41.8	363.7 ± 146.6	NS	NS	NS	KW
Membrane time constant (ms)	0.8 ± 0.1	0.6 ± 0.1	0.9 ± 0.1	NS	NS	*P* < 0.05	KW
Current amplitude (pA) evoked by pH5 acid	3460 ± 387.9	4025 ± 480.6	4877 ± 778.3	NS	NS	NS	KW
τ_desens_	188.8 ± 10.2	556.7 ± 43.8	1127 ± 100.2	*P* < 0.0001	*P* < 0.0001	NS	KW
Inhibition by MB-1	88% (8/9)	100% (3/3)	100% (2/2)	NS	NS	NS	F
Inhibition by PcTx1	33% (2/6)	80% (4/5)	NA	NS	NA	NA	F
Inhibition by APETx2	55% (5/9)	50% (1/2)	0% (0/2)	NS	NS	NS	F

*Characterization of electrophysiological properties in three types of DRG neurons with different desensitization rate (*τ*_*desens*_; rapid, intermediate and slow) of biphasic current. KW, Kruskal–Wallis test followed by Dunn’s test; ANOVA, one-way ANOVA followed by Tukey’s test; F, Fisher’s exact test; RMP, resting membrane potential; NS, not significant; NA, not available.*

**FIGURE 5:**
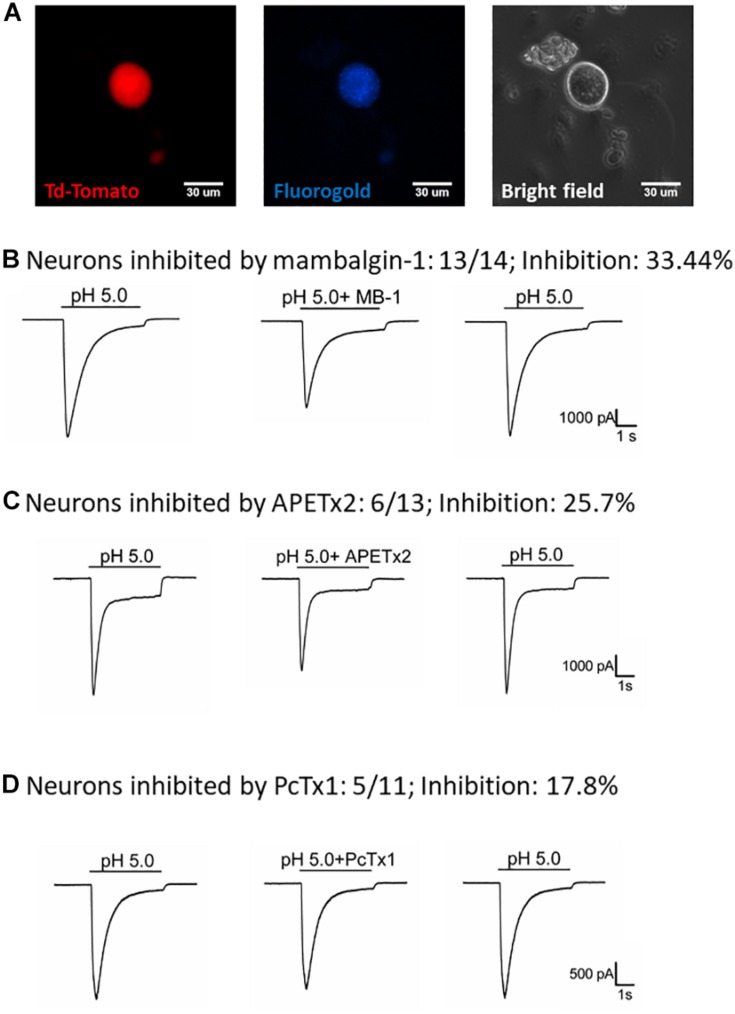
Effect of amiloride, mambalgin-1, APETx2 and PcTx1 on ASIC1b-expressing muscle afferent DRG neurons. **(A)** Whole-cell patch clamp recording on an ASIC1b-expressing DRG neuron projecting to gastrocnemius muscle labeled by fluorogold. **(B)** Mambalgin-1 (MB-1) (1 μM) inhibited acid (pH 5.0)-induced currents in 13 of 14 ASIC1b-expressing muscle afferent DRG neurons. **(C)** APETx2 (1 μM) inhibited acid (pH 5.0)-induced currents in 6 of 13 ASIC1b-expressing muscle afferent DRG neurons. **(D)** PcTx1 (100 nM) inhibited acid (pH 5.0)-induced currents in 5 of 11 ASIC1b-expressing muscle afferent DRG neurons.

To understand how the ASIC1b-expressing DRG neurons might contribute to the acid-induced mechanical hyperalgesia *in vivo*, we selectively probed the pharmacology of the *I*_ASIC_ in ASIC1b-expressing DRG neurons projecting to the gastrocnemius muscle (Figure 5A). We first tested the effect of 1 μM mambalgin-1 and found that in 13 of 14 (92.9%) ASIC1b-expressing DRG neurons, *I*_ASIC_ was partially inhibited by mambalgin-1 (inhibition rate 33.4 ± 4%) (Figure 5B). Furthermore, because even pH 6.8 acid was able to induce a robust *I*_ASIC_ (I/I_*pH*__5_ = 22.3%, mean peak current 912.6 ± 273.3 pA, *n* = 12) (Figure 4F), more sensitive ASIC subtypes such as ASIC3 or ASIC1a may support the functional ASIC channels found in ASIC1b-expressing DRG neurons.

To further characterize the involvement of other ASIC subtypes in the ASIC1b-containing channels, we used APETx2 (an antagonist of ASIC3-containing channels) and PcTx1 (an antagonist of ASIC1a-containing channels). APETx2 partially inhibited the *I*_ASIC_ (reduced 25.7 ± 5.8%) in 6 of 13 (46.2%) ASIC1b-expressing muscle afferent DRG neurons (Figure 5C), whereas PcTx1 partially inhibited the *I*_ASIC_ (reduced 17.8 ± 2.1%) in 5 of 11 (45.5%) ASIC1b-expressing DRG neurons (Figure 5D). The pharmacology results suggest that a substantial proportion of ASIC1b-expressing muscle afferent DRG neurons might contain heteromeric ASIC1b channels with ASIC1a and/or ASIC2, and also possibly ASIC3 subtypes.

**FIGURE 6:**
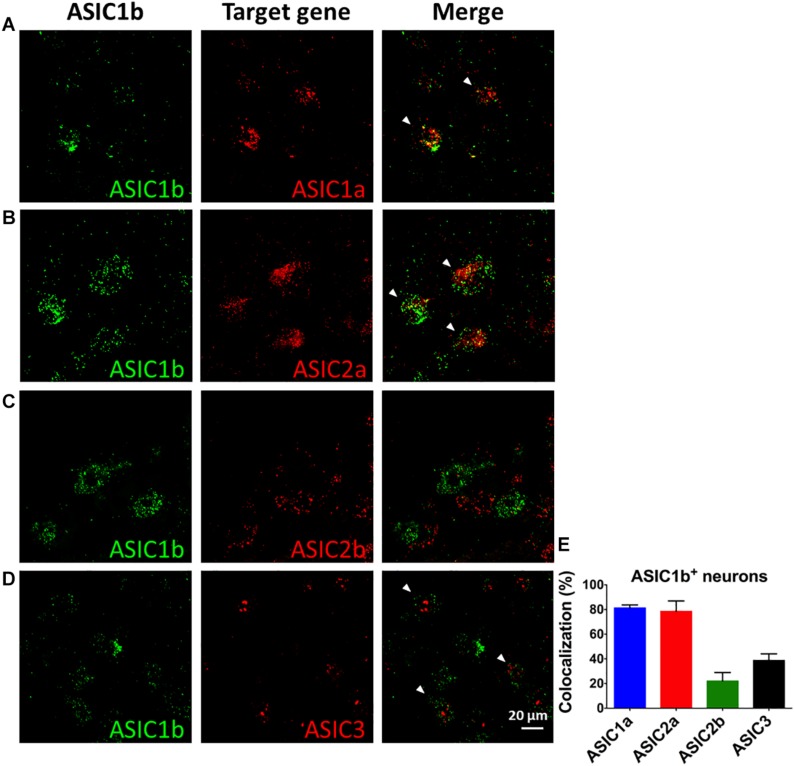
*In situ* hybridization of *Asic1a*, *Asic1b*, *Asic2a*, *Asic2b*, and *Asic3* transcripts in L4 DRG neurons. **(A–D)** Triple *in situ* hybridization of *Asic1b* with *Asic1a* **(A)** and *Asic3* **(D)** in L4 DRG. Double *in situ* hybridization of *Asic1b* and *Asic2a* **(B)**, *Asic1b* and *Asic2b* **(C)** in L4 DRG. White arrowheads indicate the co-localization of *Asic1b* with a target gene. **(E)** Quantification of the expression of *Asic1a*, *Asic2a*, *Asic2b*, and *Asic3* in *Asic1b*-positive DRG neurons (*N* = 3 for each *in situ* hybridization combination). Data are mean ± SEM.

To probe the possible ASIC composition of ASIC1b-containing channels, we used double and triple *in situ* hybridization to examine the co-localization of *Asic1b* and *Asic1a*, *Asic2a*, *Asic2b*, or *Asic3* in L4 DRG neurons (Figures 6A–D). Among *Asic1b*-positive neurons, 81, 79, 22, and 38% expressed *Asic1a*, *Asic2a*, *Asic2b*, and *Asic3*, respectively (Figure 6E). The *in situ* results were in good agreement with the electrophysiology and pharmacology data showing highly heterogeneous ASIC composition in ASIC1b-containing channels.

## Discussion

Although the expression of ASIC1b is selective in sensory neurons, ASIC1b is still a not-well-known channel as compared with other ASIC subtypes in terms of its expression, function, and contribution to pain-associated tissue acidosis (Chen et al., 1998; Diochot et al., 2012, 2016; Lin et al., 2015). In this study, we demonstrated an important role for ASIC1b-containing channels in the induction and maintenance of acid-induced chronic mechanical hyperalgesia in a mouse model of fibromyalgia. Although the exact composition of the ASIC1b-containing channels in muscle nociceptors is not known, ASIC1b might form a heteromeric ASIC channel with ASIC1a, ASIC2a, ASIC2b, or ASIC3. Pharmacological studies further validated the involvement of ASIC1a and ASIC3 in the ASIC1b heteromeric channels.

The involvement of ASIC1b in acid-induced mechanical hyperalgesia is intriguing because previous studies have shown that ASIC3 knockout would totally abolish the first acid-induced transient hyperalgesia and the development of chronic hyperalgesia induced by a second acid injection (Sluka et al., 2003; Chen and Chen, 2014; Chen et al., 2014). Of note, the first acid injection inducing transient hyperalgesia could be blocked by only APETx2 (Chen and Chen, 2014; Chen et al., 2014) and mambalgin-1 (Figure 1C) but not PcTx1 (Figure 1E). The pharmacological findings are consistent with *Asic1b* knockout results and are shown in knockout studies of *Asic1a*, *Asic3*, and *Trpv1* (Sluka et al., 2003; Chen and Chen, 2014; Chen et al., 2014). Together, ASIC1b/ASIC3 heteromeric channels would be the major player involved in the acid-induced transient hyperalgesia. However, the co-localization of ASIC1b/ASIC3 transcripts in L4 DRG neurons is relatively low (38%) as compared with ASIC1b/ASIC1a (81%) and ASIC1b/ASIC2a (79%). Further studies should probe whether the co-localization of ASIC1b and ASIC3 is present in a substantial proportion of the ASIC1b-expressing neurons projecting to gastrocnemius muscle.

Previous studies have shown the second acid injection induces no response at all in *Asic3*^–/–^ mice (Sluka et al., 2003; Chen et al., 2014). However, our *Asic1b*^–/–^ mice showed shorter duration of chronic hyperalgesia than *Asic1b*^+^*^/^*^+^ mice after dual acid injection. The fact that the second acid injection is still painful in *Asic1b*^–/–^ mice emphasizes the involvement of ASIC3 in the dual acid injection model, but ASIC3 alone is not sufficient for the development of the long-lasting hyperalgesia. Pharmacologically, inhibiting ASIC1b with mambalgin-1 (>15 pmol) in wild-type mice impaired the maintenance of chronic hyperalgesia induced by a second acid injection (Figure 2C), which was similar to the effect of inhibiting ASIC3 with APETx2 (>20 pmol) (Chen et al., 2014). These results suggest a role for ASIC1b in producing hyperalgesic priming and pain chronicity. Priming arises from neuroplasticity of injured neurons or excited neurons such that a subsequent stimulus is able to induce long-lasting pain hypersensitivity (Reichling and Levine, 2009; Sun and Chen, 2016). Here we showed that fully activated ASIC1b-containing channels in muscle afferent DRG neurons are required for the hyperalgesic priming and maintenance of chronic hyperalgesia by a repetitive intramuscular acid challenge.

Of note, the above-mentioned hyperalgesia induced by intramuscular acid injection was assessed in mouse hind paws but not the muscle. Correctly, we should name the acid-induced mechanical hyperalgesia as secondary hyperalgesia, in which the hypersensitivity responses develop in a distal site away from the noxious stimulation (Sluka et al., 2001). We did not evaluate the role of ASIC1b in the primary hyperalgesia of affected muscle in the acid-induced muscle pain model. Previous studies have shown differential role of ASIC1a and ASIC3 in the development of muscle inflammation-induced primary hyperalgesia and secondary hyperalgesia, respectively (Walder et al., 2010, 2011). On the other hand, some other studies have shown ASIC3 is involved in the development of both primary and secondary hyperalgesia in the acid-induced muscle pain model, a non-inflammatory muscle pain model (Sluka et al., 2003; Reimers et al., 2017). Nevertheless, the role of ASIC1a in the development of acid-induced primary hyperalgesia of muscle has not been tested. Since ASIC1a and ASIC1b is highly co-expressed in DRG neurons, further studies should examine the role of both ASIC1a and ASIC1b in the acid-induced primary hyperalgesia of muscle.

With the successful generation of the *Asic1b-Cre* mouse line, we have revealed the identity of ASIC1b-expressing DRG neurons. Although all ASIC1b-expressing DRG neurons are acid-sensitive, notably, 61/142 (43%) expressed an acid-induced sustained current resistant to amiloride. Excluding the 10∼20% possible mistargets of the ASIC1b-Cre line, the ASIC1b-positive neurons expressing the amiloride-resistant acid current might suggest a new direction for research into ASIC functions. These ASIC1b-expressing neurons are unlikely involved in the acid-induced hyperalgesia, because their acid-induced currents were also resistant to mambalgin-1 treatment. In some neurons, TRP channels might contribute to the amiloride-resistant acid current (Figure 4D). However, in most cases, the channel identity involved in amiloride-resistant acid current is not known. Further studies are needed to illustrate the electrophysiological and pharmacological features of these neurons as well as their physiological and pathological functions.

The other 57% of ASIC1b-expressing DRG neurons express ASIC-like currents with different electrophysiological and pharmacological properties. Their pharmacological profiles fit well with ASIC1b-containing channels, because most of the ASIC-like currents could be inhibited by mambalgin-1. However, even a high dose of mambalgin-1 (1 μM) only inhibited *I*_ASIC_ by 33.4 ± 4%, which suggests the contribution of other mambalgin-1–insensitive ASIC channels in ASIC1b-expressing DRG neurons. Some of these ASIC-like currents could be partially inhibited by PcTx1 and APETx2, so ASIC1a and ASIC3 might form heteromeric channels with ASIC1b in these ASIC1b-expressing DRG neurons. Previous studies have shown the inhibiting effects of mambalgin-1 on ASIC1a, ASIC1b, ASIC1a + ASIC1b, ASIC1a + ASIC2a, and ASIC1a + ASIC2b channels but not ASIC2a, ASIC3, ASIC1a + ASIC3, and ASIC1b + ASIC3 channels (Diochot et al., 2012). In our *in situ* hybridization data, we found that ASIC1b-expressing neurons largely co-expressed with ASIC1a and ASIC2a, for a possible involvement of various heteromeric ASIC channels in the *I*_ASIC_.

These ASIC1b-expressing DRG neurons with *I*_ASIC_ were medium- to large-sized neurons with diameters ranging from 30 to 55 μm. The heterogeneity of the ASIC1b-expressing DRG neurons suggest that they might play multiple roles in different physiological and pathological contexts, because ASICs are proposed as dual-function proteins for acid- and mechanosensing (Cheng et al., 2018; Chuang et al., 2018; Lin et al., 2018). Especially, the expression of ASIC1b in large-diameter DRG neurons might implicate its role in proprioception similar to ASIC3 (Lin et al., 2016).

In conclusion, ASIC1b plays an important role in acid-induced mechanical hyperalgesia. The ASIC1b-containing channels are highly heterogeneous and might contain different combinations of ASIC subtypes in different ASIC1b-expressing DRG neurons. This study provides a new direction for future research into pain-associated tissue acidosis and ASIC-mediated somatosensory functions.

## Data Availability Statement

All datasets generated for this study are included in the article/supplementary material.

## Ethics Statement

The animal study was reviewed and approved by Institutional Animal Care and Utilization Committee of Academia Sinica.

## Author Contributions

C-TC conducted and analyzed the electrophysiology and *in situ* hybridization experiments. SF performed and analyzed the von Frey behavior experiments. C-HL assisted and provided the experimental design in von Frey behavior experiments. Y-CC performed and analyzed the ASIC expression experiments. S-HL generated the ASIC1b-cre and ASIC1b knockout mice. C-TC and C-CC collected, organized, and interpreted the result and prepared the manuscript.

## Conflict of Interest

The authors declare that the research was conducted in the absence of any commercial or financial relationships that could be construed as a potential conflict of interest.
